# Harnessing Innovative Technologies to Train Nurses in Suicide Safety Planning With Hospitalized Patients: Protocol for Formative and Pilot Feasibility Research

**DOI:** 10.2196/33695

**Published:** 2021-12-15

**Authors:** Doyanne Darnell, Patricia A Areán, Shannon Dorsey, David C Atkins, Michael J Tanana, Tad Hirsch, Sean D Mooney, Edwin D Boudreaux, Katherine Anne Comtois

**Affiliations:** 1 Department of Psychiatry & Behavioral Sciences University of Washington Seattle, WA United States; 2 Department of Psychology University of Washington Seattle, WA United States; 3 Social Research Institute University of Utah Salt Lake City, UT United States; 4 College of Arts, Media, and Design Northeastern University Boston, MA United States; 5 Department of Biomedical Informatics and Medical Education University of Washington Seattle, WA United States; 6 University of Massachusetts Medical School Worcester, MA United States

**Keywords:** suicide prevention, hospital, training, e-learning, artificial intelligence, implementation science, user-centered design, task-shifting, quality assessment, fidelity

## Abstract

**Background:**

Suicide is the 10th leading cause of death in the United States, with >47,000 deaths in 2019. Most people who died by suicide had contact with the health care system in the year before their death. Health care provider training is a top research priority identified by the National Action Alliance for Suicide Prevention; however, evidence-based approaches that target skill-building are resource intensive and difficult to implement. Advances in artificial intelligence technology hold promise for improving the scalability and sustainability of training methods, as it is now possible for computers to assess the intervention delivery skills of trainees and provide feedback to guide skill improvements. Much remains to be known about how best to integrate these novel technologies into continuing education for health care providers.

**Objective:**

In Project WISE (Workplace Integrated Support and Education), we aim to develop e-learning training in suicide safety planning, enhanced with novel skill-building technologies that can be integrated into the routine workflow of nurses serving patients hospitalized for medical or surgical reasons or traumatic injury. The research aims include identifying strategies for the implementation and workflow integration of both the training and safety planning with patients, adapting 2 existing technologies to enhance general counseling skills for use in suicide safety planning (a conversational agent and an artificial intelligence–based feedback system), observing training acceptability and nurse engagement with the training components, and assessing the feasibility of recruitment, retention, and collection of longitudinal self-report and electronic health record data for patients identified as at risk of suicide.

**Methods:**

Our developmental research includes qualitative and observational methods to explore the implementation context and technology usability, formative evaluation of the training paradigm, and pilot research to assess the feasibility of conducting a future cluster randomized pragmatic trial. The trial will examine whether patients hospitalized for medical or surgical reasons or traumatic injury who are at risk of suicide have better suicide-related postdischarge outcomes when admitted to a unit with nurses trained using the skill-building technology than those admitted to a unit with untrained nurses. The research takes place at a level 1 trauma center, which is also a safety-net hospital and academic medical center.

**Results:**

Project WISE was funded in July 2019. As of September 2021, we have completed focus groups and usability testing with 27 acute care and 3 acute and intensive care nurses. We began data collection for research aims 3 and 4 in November 2021. All research has been approved by the University of Washington institutional review board.

**Conclusions:**

Project WISE aims to further the national agenda to improve suicide prevention in health care settings by training nurses in suicide prevention with medically hospitalized patients using novel e-learning technologies.

**International Registered Report Identifier (IRRID):**

DERR1-10.2196/33695

## Introduction

### Background

Suicide is the tenth leading cause of death in the United States, with >47,000 deaths in 2019 [1]. Most people who died by suicide had contact with the health care system in the year before their death, both in general medical and in acute care settings, and frequently for reasons other than behavioral health [2-4]. Therefore, to better reach patients in general medical settings, the National Action Alliance for Suicide Prevention designed an agenda that prioritizes training a wide range of health care providers in suicide prevention [5]. There is increasing awareness of *occult* or hidden suicidality or suicide risk among hospitalized patients presenting for medical reasons, surgeries, or traumatic injury. When implemented in acute medical care settings, universal suicide screening programs routinely identify patients at risk for suicide who would otherwise have gone unnoticed [6-10]. For instance, a study conducted with patients seen in emergency departments (EDs) across 8 hospitals and 7 states observed twice the rate of suicide risk detection after implementing universal screening [11]. Many medically hospitalized patients are known to have risk factors for suicidality, such as high rates of behavioral health conditions [12-14]. Hospitalization experiences, such as being admitted to an intensive care unit, are known to increase the risk factors for suicidality, including posttraumatic stress disorder (PTSD) and depression [15], and reasons for hospitalization, such as traumatic injury, are known to place patients at a greater risk of suicide following discharge [16]. Therefore, the Joint Commission, which provides oversight, standards, and guidelines for health care organizations nationally, recommends screening for suicide risk among medically hospitalized patients and mitigating risk with strategies such as suicide safety planning [17]. To support patients identified as at risk of suicide in these hospital settings, we aim to develop Project WISE (Workplace Integrated Support and Education), which includes research to develop an e-learning training in suicide safety planning enhanced with novel skill-building technologies that can be integrated into the routine workflow of nurses serving patients hospitalized for medical or surgical reasons or traumatic injury.

### Continuing Education for Health Care Providers in Suicide Prevention

There has been a proliferation of e-learning and in-person continuing education programs designed to train a workforce that has otherwise been naïve to suicide prevention. State licensing boards are increasingly requiring health care providers to complete several hours of suicide-prevention continuing education at least once, if not routinely, every several years [18,19]. *Gold standard* evidence-based training approaches aim to improve both knowledge and skills about suicide and how to intervene and include some form of didactic training (in-person or web-based) with a demonstration or modeling of suicide prevention skills, opportunities to practice these skills, and expert coaching and feedback on practicing these skills [20-22]. Many also target attitudes for increasing willingness and motivation to engage in suicide-prevention activities, given that stigma, anxiety, and unhelpful myths about suicide are common among the general population and professionals [23,24]. In addition, evidence-based continuing education harnesses what is known about how adults learn in real-world environments, such as allowing clinicians to be self-directed and solve real-life problems [25,26].

Although evidence-based training and suicide-prevention continuing education approaches exist (eg, the Question, Persuade, and Refer Gatekeeper training and LivingWorks Applied Suicide Intervention Skills training for safety planning [27,28]), peer-reviewed research on training outcomes is limited with regard to the impact of training on skills [29-31]. Even less is known about how well the trainees apply and maintain these skills in routine intervention delivery; this is a concern, as skills frequently drift from higher to lower quality following training, and this drift is a critical barrier to the sustainment of evidence-based practices [32].

### Using Technology to Enhance the Scalability and Sustainability of Continuing Education in Suicide Prevention

Our research is designed to address 2 common and related barriers to the widespread implementation of effective continuing education and the transfer of what was learned from the training to practice in health care settings. The first is the need for efficient and low-cost training that targets skill-building for suicide-prevention activities. The gold standard methods for training require considerable provider and expert trainer time for didactics, skills practice, fidelity or quality assessment, and feedback or coaching sessions [22,32,33]. Therefore, continuing education generally requires taking large amounts of time away from work at considerable financial costs to the trainee and their employer. Busy providers and organizations with limited access to resources for training may opt to engage in briefer and less costly training such as web-based didactics [34], although didactics alone are insufficient for provider behavior change [22].

The second barrier is the lack of efficient tools to evaluate the quality of trainee suicide-prevention skills, both for training and skill maintenance purposes. Quality refers to the extent to which an intervention is delivered well enough for it to achieve its expected effects [35]. Traditional methods of quality assessment for psychosocial interventions are not scalable in routine training and quality improvement contexts [22,32,33]. These methods rely primarily on humans to convert complex qualitative data (eg, transcripts or observations of recorded interactions) into simplified and summative quantitative information [36], which is labor-intensive and expensive, requiring a well-trained and reliable coder to code samples of skills practice.

Advances in technology are promising for reducing the barriers to implementing effective, evidence-based training and reaching the full spectrum of health care providers who may engage patients in suicide-prevention activities. Although web-based learning platforms with didactic content are routinely available for provider training in psychosocial interventions [34], more recent innovations using computer technologies allow the trainees to practice relevant skills using simulated training environments, replacing human effort with computer technology to facilitate skill practice with feedback on their performance [37-41]. We designed Project WISE to develop and implement web-based, technology-enhanced suicide-prevention training for nurses serving patients hospitalized for medical or surgical reasons or traumatic injury. The training is designed to be effective, efficient, and ultimately low-cost. To be most widely scalable, the training will be flexibly designed with components that can be integrated into a routine workflow in a clinical setting.

### Project WISE: Technology-Enhanced Training in Suicide Safety Planning

The Joint Commission recommends suicide safety planning for patients in hospital settings at risk of suicide. Suicide safety planning can be a brief, 30- to 45-minute intervention in which a provider works collaboratively with a patient to identify a multistep plan for coping with suicidal thinking and urges to prevent suicidal behavior [42,43]. Coping strategies include ways to distract from suicidal thinking and seek help from others, both through social support and professional help. A provider helps the patient identify experiences, thoughts, or feelings that commonly lead to suicidal thoughts so that the patient knows when to engage in the safety plan. Safety plans also include strategies for limiting access to lethal ways to die (eg, locking firearms). Suicide safety planning requires providers to effectively use general counseling skills [44], including empathic listening to understand patients’ experiences, reflection of this understanding, and the ability to work collaboratively with patients to generate useful and relevant safety strategies. Research shows that collaboratively developed safety plans are of higher quality [45,46]. In a recent study of veterans seeking emergency services for suicidality, suicide safety planning was associated with a 50% reduction in suicidal behavior over 6 months [47]. Safety planning with active US army soldiers seeking emergency behavioral health care was effective in reducing suicide attempts as compared with contracting to not engage in suicidal behavior (5% vs 19%) [43].

**Figure 1:**
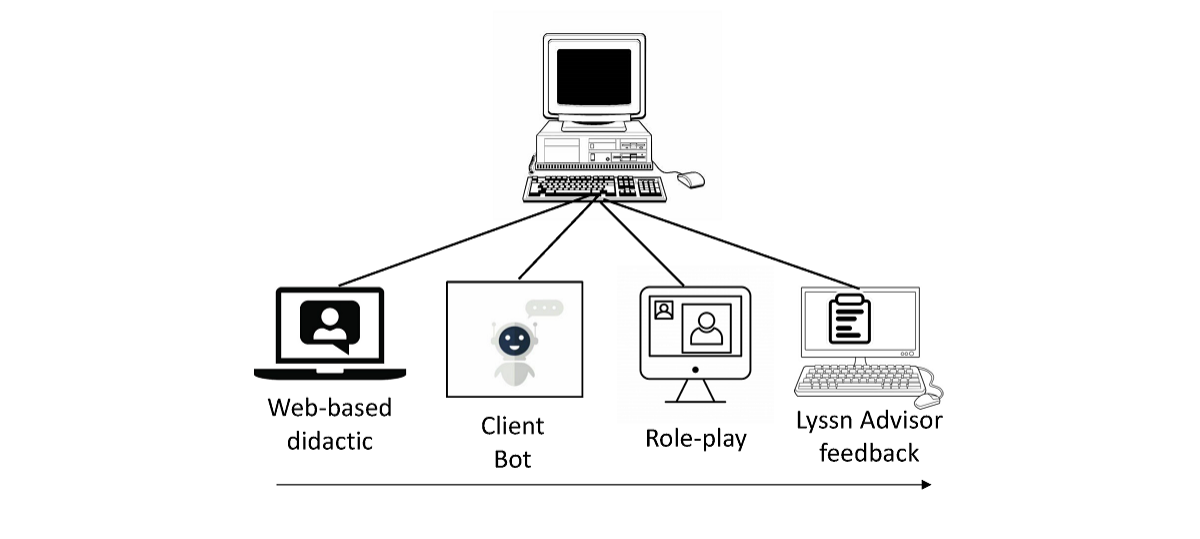
Project WISE (Workplace Integrated Support and Education) e-learning training in suicide safety planning. The webinar includes didactic training and demonstration of safety planning. Client Bot allows for practice with feedback in counseling microskills. Role-play includes practice in doing safety planning with a patient actor. Lyssn Advisor feedback includes review of computer-generated feedback for empathy and collaboration skills and a recording of the role-play.

Project WISE’s e-learning training in suicide safety planning includes several components consistent with adult learning principles and effective practices for training in evidence-based interventions (Figure 1). The training will take 2.5 to 3 hours and begin with a web-based didactic training that includes a demonstration of safety planning. Much of the content for the didactic is publicly available from the Joint Commission resources on suicide safety planning, and other content is crafted for nurses by the first author (DD). The 1-hour didactic training may be completed over ≥1 session, with the intention of having it done within 1 week.

Following the didactic training, nurses will complete a 30-minute role-play practice with a conversational agent called Client Bot, developed by Lyssn. Trainees may complete this training in one sitting or across multiple sessions. The Client Bot technology uses machine learning and artificial intelligence to simulate interactions with a patient in text format, providing the opportunity for trainees to practice general counseling microskills (eg, reflective statements of what a patient says or means and open questions to elicit a patient’s perspective and interests) and receive real-time feedback on performance and coaching on the use of these skills. Microskills training is known to be an effective method for improving counseling skills during counseling sessions [48], and Client Bot has successfully trained novice counselors to generate empathic reflective statements and ask open-ended questions [39]. Practice with the Client Bot is also expected to help increase confidence in inquiring about and discussing suicidality with simulated and real patients.

Following the didactic webinar and microskills practice, trainees will practice suicide safety planning with a human actor role-playing a medically hospitalized patient at risk of suicide. Such simulated patient interactions are common in medical training and clinical skills evaluation [49]. The 30-minute role-play will be completed using videoconferencing software and will be recorded. The recording will be processed through a system called Lyssn Advisor, developed by Lyssn, that uses speech signal, natural language processing, and machine learning to first convert the audio content to a transcript and then assess the provider’s quality of general counseling skills based on the transcript text [40,50]. The feedback will provide information on the quality of general counseling skills, such as using a collaborative, empathic style with the patient. The Lyssn Advisor system generates a confidential feedback report accessible via the internet at the trainee’s convenience. The trainee will be encouraged to spend approximately 30 minutes reviewing their report and parts of their role-play and to use the feedback to inform their counseling style in future simulated or real patient suicide safety planning interventions. Trainees will be welcome to spend more time with the Client Bot if they would like to practice microskills after receiving computer-generated feedback through the Lyssn Advisor system. In routine deployment of the training, we expect that trainees will have the option to engage in additional role-plays and obtain feedback on these role-plays.

### Project WISE Research Aims

**Figure 2:**
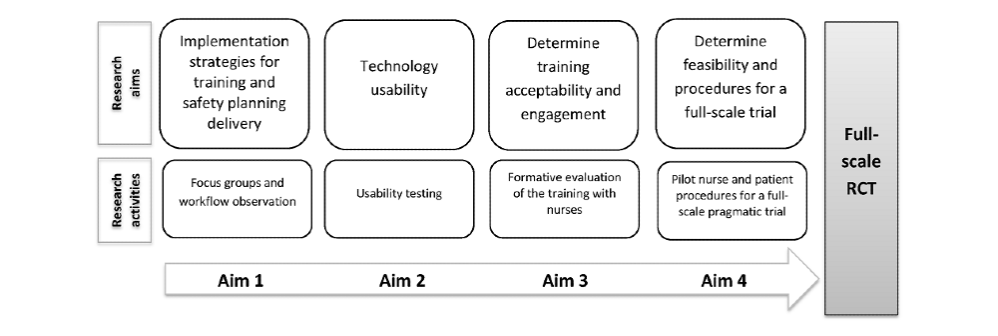
Project WISE (Workplace Integrated Support and Education) research aims. RCT: randomized controlled trial.

Much remains to be known on how best to integrate the novel conversational agent (Client Bot) and automated feedback (Lyssn Advisor) technology for skills training into continuing education for health care providers and how to optimize the transfer of skills learned for the greatest impact on patient outcomes. Project WISE includes developmental and pilot research to inform the refinement and deployment of e-learning training as well as the design of a future large-scale pragmatic trial. The future trial will examine whether hospitalized medical, surgical, or traumatically injured patients at risk of suicide have better suicide-related postdischarge outcomes if admitted to a unit with nurses trained using technology-enhanced training in suicide safety planning as compared with those admitted to a unit with untrained nurses. Our interdisciplinary team will use concepts and methodology from implementation science, adult learning theory, user-centered design, and suicide-prevention research to conduct the 4 research aims (Figure 2): (1) identify strategies for the implementation and workflow integration of both the technology-enhanced training as well as the delivery of suicide safety planning with patients, (2) adapt the existing Client Bot and Lyssn Advisor technologies to train nurses in general counseling skills for use in suicide safety planning, (3) conduct a formative evaluation of the training with nurses to assess training acceptability and engagement with the training components and technologies and inform the iterations of the training, and (4) assess the feasibility and pilot procedures for a cluster randomized trial evaluating the impact of the training on patient outcomes.

## Methods

The methods specific to each study aim have been described in the following sections. All procedures were approved by the University of Washington institutional review board.

### Setting and Population

The Project WISE research takes place at a level 1 trauma center, which is also a safety-net hospital and academic medical center. Nurses there are required to complete 6 hours of suicide-prevention training at least once for state licensure and universally screen all patients admitted to the ED and inpatient units for suicidality. The Columbia Suicide Severity Rating Scale (C-SSRS) triage version [51] was adopted by the hospital and implemented as part of the universal screening protocol in the ED in October 2019 and on inpatient units in July 2020. The usual care for patients screening as high risk includes suicide precautions such as ensuring that the environment is safe from lethal means, having a patient monitor sit with the patient, and notifying the medical team, who would request a consult from the hospital psychiatry service. Low- or moderate-risk patients are provided suicide-prevention resources at discharge (eg, crisis line) and may request to see a hospital social worker. Units serving medical, surgical, and traumatic injury patients will be recruited to participate. Unit managers will help facilitate the recruitment of nurses and have agreed to have the research team recruit patients. On the basis of prior research with local and similar trauma centers, it is anticipated that nurses will predominantly identify as female and White [52] and that the patients will predominantly identify as male [53] and White [54].

### Aim 1: Focus Groups and Contextual Inquiry

#### Design

Aim 1 combines implementation science and user-centered design methods to collect qualitative and observational data to identify the strategies for implementing both nurse training and the transfer of learning from training simulations to the conduct of suicide safety planning with actual patients. Aim 1 includes the conduct of 3 to 6 focus groups with nurses serving medical, surgical, or trauma inpatients (n=4-6 per group) to assess the barriers to and facilitators of engaging in the training and delivery of suicide safety planning with hospitalized patients using the Theoretical Domains Framework (TDF) [55]. The TDF synthesizes 33 theories with relevance to provider behavior change associated with implementing an evidence-based practice, resulting in 14 domains covering individual-, setting-, and organizational-level variables that are relevant to both training engagement and training transfer [56,57].

Aim 1 also includes the user-centered design method of contextual inquiry and task analysis, with 3 nurses to observe and inquire about actual nursing workflows [58]. Task analysis includes shadowing nurses as they demonstrate their current practices, including preparing and documenting patient encounters, their information technology interaction, and their workflow. Data collected will include field notes of observations and audio recordings of real-time discussions with nurses on workflow integration of both the training components and the delivery of safety planning with patients. Real-time questions will further assess the TDF constructs (eg, emotions and beliefs about capabilities).

In addition to these research activities, members of the team will meet with various hospital stakeholders for guidance and input on Project WISE, suicide-prevention initiatives, and the implementation of research activities. A key stakeholder group is a committee that approves and advises research projects that involve nurses and nursing services. Decisions about nursing research also occur at the unit level; therefore, the team will engage unit nurse managers in planning for research activities.

#### Plan of Analysis

Focus groups will be audio recorded and transcribed for analysis, which will include a qualitative content analysis [59] to identify the presence, absence, and specific characterizations of potential implementation barriers and facilitators based on focus group data. A priori themes will be identified, and an initial coding scheme will be developed based on TDF constructs and then refined after reviewing each transcript. The transcripts will be coded by 2 coders. Discrepancies will be resolved by consensus and estimates of interrater reliability will be calculated [60]. The findings may be presented through narratives, tabular representations of themes with illustrative quotes, and thematic counts. The Atlas.ti (ATLAS.ti Scientific Software Development, GmbH) computer program [61] will assist with the analysis [62,63]. The contextual inquiry will result in task flow diagrams of key user processes, which are detailed, visual depictions of the steps a user takes to complete a task with the technology, and identify a workflow for engaging patients in safety planning.

### Aim 2: Usability Testing

#### Design

A user-centered design methodology will be used to update the user interfaces of both the Client Bot and Lyssn Advisor technologies for nurse end users and inform the instructional support provided to nurses regarding how to use the technology. We will conduct usability testing with nurses, followed by the completion of a brief questionnaire to obtain their feedback on the novel technologies. The 3 nurses will interact with the Client Bot and the Lyssn Advisor feedback report. For Client Bot, a 15-minute session will include text-based *chatting* with a simulated, suicidal patient. Nurses will be instructed to use general counseling microskills, including asking open-ended questions and making empathic reflections on what the client has said. Throughout the chat session, nurses will be provided immediate, adaptive feedback when they use these skills and encouragement to do so when they do not. The first author will observe the nurses interacting with the Client Bot system and record their experiences. Nurses will also be asked to verbalize their thought process as they interact with the technology (eg, *concurrent think-aloud protocol* [64]). These nurses will also be asked to interact with a sample version of the Lyssn Advisor feedback system based on a sample suicide safety planning intervention, again using the *concurrent think-aloud protocol*, and providing feedback on the provided information and the manner in which it is presented. Usability will also be measured with the System Usability Scale (SUS) [65], a 10-item Likert scale survey that has been widely used in usability research as a measure of user satisfaction.

#### Plan of Analysis

The research team will make observations during the usability testing sessions and use video recordings of the sessions. The team will document misunderstandings, frustrations, technology errors or problems, participant errors, what went well in using the technology, successes in using the system, and suggestions made by the participants. Usability will be indicated with a target SUS score of 68 out of 100. Changes will be made to the user interface to achieve sufficient user satisfaction ratings based on the feedback from usability testing.

### Aim 3: Formative Evaluation of the Training

#### Design

For the formative evaluation of e-learning training with nurses, we will assess the acceptability of and engagement with the training components and technologies using longitudinal survey research, observation of nurse performance in suicide safety planning, and end-of-evaluation focus groups. The evaluation will be registered at ClinicalTrials.gov.

#### Procedures

A total of 20 nurses from the participating acute or intensive care units will be recruited by the research team to participate. They will be invited to complete all the training components (Figure 1), including the web-based didactic training and demonstration of suicide safety planning, practice of counseling microskills with the Client Bot, a 30-minute role-play practice with a patient actor, and computer-based feedback on this practice through the Lyssn Advisor system. Completion of all training components is expected to occur within 1 month; however, nurses may access these materials at any time during the 6-month study follow-up period.

Evaluation activities will include a series of surveys (before training, after training, and at the 6-month follow-up) and an end-of-study focus group to collect data on the acceptability of the training and technology and barriers to and facilitators of the implementation of the training and suicide safety planning delivery. In addition, nurses will complete a series of standardized patient role-plays to assess their skills in suicide safety planning before and after interacting with each of the novel technologies. A follow-up role-play will occur 6 months after completing all the training components.

#### Measures and Variables

##### Demographics

In the baseline survey, the nurses will be asked about their demographics (eg, gender, race or ethnicity, and age), length of their current employment, training background, and experience with suicide prevention.

##### Acceptability of the Training

In follow-up surveys, the nurses’ training and technology acceptability will be assessed using the SUS [65] and open-ended questions to elaborate on what contributes to their responses on the SUS. The SUS has 10 statements that are responded to on a Likert-type scale ranging from 0 (strongly disagree) to 4 (strongly agree). The scores range from 0 to 100, with higher scores indicating greater acceptability (usability). The SUS has demonstrated good internal consistency, reliability, and concurrent validity with other usability measures [66].

##### Training Completion and Technology Use

Training completion and use of the technology will be assessed with self-report surveys after training and at the 6-month follow-up. This will include questions about whether and when the participants used the Client Bot and Lyssn Advisor feedback system and how they used it in their training and practice. The didactic portion will have embedded knowledge questions to track the completion of didactic content. Use data will also be collected by the Client Bot and Lyssn Advisor systems, which will comprise how often the nurses accessed the program, times of day for use, how long the nurses spent on the program, and the features that they used.

##### Motivation for Training and Delivery of Suicide Safety Planning

A measure of nurse motivation to use the training materials, including the technology, will be asked at each survey time point. This will include variables such as the nurses’ interest in the material, technology, and willingness to persist when challenged by practicing skills and receiving corrective feedback. The motivation to use general counseling skills to conduct collaborative safety planning with patients (ie, transfer of training [56]) will be assessed by adapting measures from the TDF (eg, beliefs about capabilities and consequences, behavioral intentions, and negative emotions) [55].

##### Suicide Safety Planning Quality

The quality of the use of general counseling skills and safety planning skills will be assessed using standardized patient role-play assessments. Nurses will complete four 30-minute role-plays over the course of the evaluation. Role-play scenarios will be developed that allow nurses to practice the full range of safety planning skills with a willing, hospitalized patient who has previously experienced a suicidal crisis. A role-play will be used as part of the training and for providing evaluation data as it will be uploaded to the Lyssn Advisor system for nurses to review their performance. All role-plays will be conducted by a patient actor via video web-conferencing software, recorded, and assessed for general counseling skill quality using the Lyssn Advisor system. The Lyssn Advisor system codes for provider empathy and collaboration, consistent with the Motivational Interviewing Skill Code [67].

The postwebinar, posttraining, and 6-month follow-up role-plays will be assessed for quality of the safety planning intervention by the first author using the Safety Planning Intervention Rating Scale [68]. The scale scores range from 0 to 20, with higher scores indicating greater quality of safety planning (eg, whether the key components were done and how well they were done). Self-reported perception of skills will be assessed via surveys at baseline, after training, and at 6 months after training using methods modified from previous research on training clinicians in evidence-based psychotherapy [69]. Specifically, nurses will report their self-perceived level of skill on a Likert-type scale for engaging in components of the safety planning intervention covered in the Safety Planning Intervention Rating Scale.

##### Implementation Barriers and Facilitators

Implementation barriers and facilitators will be assessed for both engaging in training and using the skills learned with patients via nurse surveys at baseline, after training, 6 months after training, and at end-of-study focus groups. Questions will be designed using the TDF (eg, beliefs about consequences, role and identity, and environmental context and resources).

### Plan of Analysis

Quantitative data will be viewed graphically and analyzed descriptively. Qualitative open-ended responses to questions will be summarized into common themes, or their content will be analyzed as appropriate. Acceptability of the training will be indicated by a target score of 68 out of 100 on the SUS. We will observe the rates of training completion per training component, and follow-up assessments will explore reasons for noncompletion based on self-report. The findings will be used to inform iterations to improve the acceptability of and engagement with the training. Focus groups will be content analyzed using the same methods as described for aim 1 and will be used to inform the implementation of the training and suicide safety planning with patients in a future trial.

### Aim 4: Pilot Procedures and Feasibility Assessment for Conducting a Full-Scale Pragmatic Trial

Aim 4 will inform the design of a cluster randomized trial, including whether to use a parallel or stepped-wedge design [70], measurement approaches, and sample size determination to obtain adequate statistical power.

#### Design

**Figure 3:**
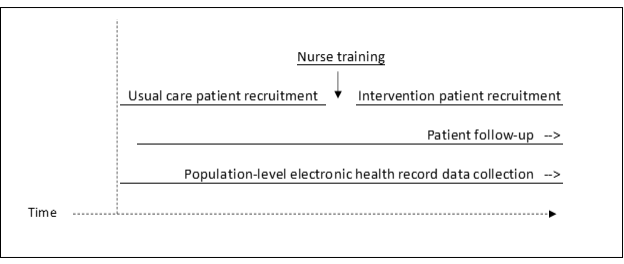
Anticipated timing of patient enrollment, nurse training, and data collection at the unit level for a future full-scale cluster randomized clinical trial evaluating the impact of the training on patients’ suicide-related outcomes.

In the future full-scale trial, randomization will occur at the hospital unit level, and patient recruitment will occur for a period before and after the training of nurses in the unit on suicide safety planning, resulting in usual care and intervention group samples, respectively (Figure 3). Nurse training outcomes will also be assessed longitudinally in the future trial, and aim 3 provides pilot data related to the feasibility of retention and the assessment of training outcomes for nurses.

We will pilot the longitudinal follow-up of a sample of patients identified as at risk of suicide based on the C-SSRS triage version, including the assessment of feasibility for recruitment, retention, and self-report of suicide-prevention service use and suicide-related outcomes over 6 months. We will also pilot the collection of electronic health record (EHR) data linked to patients to observe what is documented in the EHR regarding suicide-prevention services received while at the hospital and patient outcomes (eg, readmission for suicidal ideation or behavior) over the follow-up period. Data may be abstracted directly from the EHR by the research team or collected from a data warehouse.

There are limited data on suicide screening results for patients hospitalized for medical or surgical reasons or traumatic injury. A study implementing the C-SSRS with hospitalized trauma patients observed a 4% rate of positive screens [10]. With an estimated 6000 trauma patients in the study hospital per year [71], we might expect 20 potential participants per month on trauma units alone. To better estimate potential recruitment rates, we will collect deidentified population-level data for C-SSRS screening in acute and intensive care over a 1-year period. EHR data collected naturalistically in the course of clinical care are commonly harnessed in pragmatic trial research [72]. For instance, it is possible to collect data on suicide-related hospital visits, such as admissions for self-inflicted injuries or suicide attempts. Such outcomes can be collected for the entire population of patients seen during a specific time frame and could be used to provide a population-level estimate of the effect of introducing suicide safety planning training on hospital units, as opposed to relying solely on an estimate from a subsample of the population followed over time. Therefore, we will develop and pilot procedures for collecting population-level deidentified EHR data to pragmatically assess suicide-prevention service delivery for hospitalized patients (eg, documentation of a psychiatry consultation for suicide risk, suicide risk assessments, and documentation of safety plans) and suicide-related patient outcomes (eg, ED admissions for suicidal ideation and hospital admissions for self-inflicted injury).

#### Procedures

A total of 40 adults aged ≥18 years admitted to the hospital for medical or surgical reasons or traumatic injury will be recruited based on having a positive C-SSRS triage screener result (moderate or high) as recorded in the EHR. Exclusion criteria will be patients unable to consent to the research, such as because of cognitive impairment or active psychotic symptoms, prisoners, and non–English speaking patients. Enrolled patients will complete a baseline survey and provide detailed contact information for follow-up purposes. At the end of the enrollment process, patients will be provided with national crisis line resources. All participants will receive usual care for suicide risk, which may include the removal of lethal means from a patient’s hospital room, designation of a person to sit with and monitor the patient for safety purposes, or referral to the hospital-based psychiatry consultation service for evaluation and referral.

Patients will complete the study measures via surveys at baseline, 1 month, and 6 months. Surveys may be completed via self-report or interviews with the research staff. To address safety concerns, the surveys will incorporate procedures from the University of Washington risk assessment protocol [73]. For self-report surveys, certain responses indicative of active suicidal ideation will trigger contact from the research staff to follow-up on safety concerns. EHR data relevant to the injury event and initial and follow-up clinical care will also be collected, such as C-SSRS screening results, documentation of suicide-prevention interventions in the hospital, International Classification of Diseases codes for suicidal ideation and self-inflicted injury associated with their inpatient hospitalization, and readmissions to the hospital for suicidal ideation or behavior during the 6-month follow-up period. Subsequent visits to any ED in the state will also be observed using the ED information exchange system [74].

Population-level deidentified data for medical, surgical, or traumatic injury patients will be collected from the hospital data warehouse and will include the collection of state-level death records data that are integrated with these EHRs. The team will identify potential sources of clinical data related to suicide such as reasons for patient admission, services delivered, and patient clinical characteristics and work with data warehouse biomedical informaticians to locate and pull these data from the warehouse.

#### Measures and Variables

##### Demographics and Clinical Characteristics Related to Hospitalization

Patients will complete self-report demographic questions on information such as their gender, race or ethnicity, age, and socioeconomic status. Clinical characteristics of their hospitalization will be abstracted from the EHR regarding suicide screening results, services received during the hospital stay, International Classification of Diseases-10 codes associated with their stay, and admission diagnosis or reason for hospitalization.

##### Columbia Suicide Severity Rating Scale

The C-SSRS triage version [51] will be asked for each patient upon admission to the study hospital’s ED or inpatient unit. There are 6 yes or no questions to assess the past month’s suicidal ideation severity and lifetime and the past 3 months’ preparatory behavior or suicide attempt. On the basis of these responses, patients will be determined to be at low risk, moderate risk, or high risk. The presence of a detailed plan, suicidal intent, preparatory behavior, or suicide attempt in the past 3 months will result in patients being considered at high risk. The C-SSRS is recommended as a screening tool by the Joint Commission [75].

##### Hospital-Based Suicide-Prevention Services

We will assess the suicide-prevention activities conducted during the patient’s hospitalization via EHR documentation and patient self-report at the 1-month assessment. Patients will also self-report their perception of what was helpful or not helpful with regard to these activities at both follow-up assessments.

##### Suicide-Related Outcomes

Patients will self-report suicidal ideation and behavior using the Self-Injurious Thoughts and Behaviors Interview–revised (SITBI-R) [76]. EHR and ED readmission data for visits related to suicidal ideation and self-inflicted injury will also be assessed.

We will use a subset of items from the SITBI-R to assess suicide ideation, suicide planning, preparatory behaviors, suicide threats and gestures, aborted suicide attempts, interrupted suicide attempts, and suicide attempts [76]. Questions regarding the age of onset, frequency of thought or behavior, duration of thought or behavior, urge or intensity, and future likelihood of thought or behavior engagement will be asked. Items responses include Likert-type ratings and open-ended and multiple-choice options. The measure has demonstrated good test-retest reliability and convergent validity with an existing measure for use both as an interview assessment and self-report in a web-based survey format. The SITBI-R will be asked at all time points.

Patients will self-report self-efficacy to avoid suicidal behavior at each time point using the Suicide-Related Coping Scale [77]. This scale includes 17 items rated on a Likert-type 5-point scale ranging from 0 (strongly disagree) to 4 (strongly agree). The items assess the knowledge of and confidence in using internal coping strategies and external resources to manage suicidal thoughts and urges to decrease risk and avert suicidal crises (eg, when I am suicidal, I know of things to do by myself that help me feel less suicidal; I know which friends or family members to contact to help take my mind off my suicidal feelings).

##### Behavioral Health Service Use

Patient behavioral health service use will be collected via self-report and EHRs, when available. Questions will assess whether, how often, and what types of services patients used for reasons of mental health and substance use or addiction. Lifetime and previous month assessments will occur at the 1-month follow-up, and previous 6 months’ assessment will occur at the 6-month follow-up.

##### Behavioral Health Symptoms

Patients will be asked about depression and anxiety at each assessment. Self-reported depression symptoms will be assessed using the Patient Health Questionnaire 9-item version [78], a well-validated and reliable measure that assesses the severity of depressive symptoms according to the *Diagnostic and Statistical Manual of Mental Disorders, Fifth Edition* (*DSM-5*) criteria [79]. Items are rated on a scale from 0 (not at all) to 3 (nearly every day) and summed for a total score, with higher scores indicating more depression. A score of ≥10 indicates clinically significant depression symptoms. Self-reported anxiety symptoms will be assessed using the General Anxiety Disorder 7-item scale [80], a well-validated and reliable measure that assesses the severity of generalized anxiety based on the *DSM-5*. Items are rated on a scale from 0 (not at all) to 3 (nearly every day) and summed for a total score, with higher scores indicating greater levels of anxiety. A score of ≥10 indicates clinically significant generalized anxiety symptoms.

Patients will be asked about their trauma history and posttraumatic stress symptoms at the 1-month follow-up and posttraumatic stress symptoms at the 6-month follow-up. Patients will self-report on [81] whether they have experienced the 16 events in the Life Events Checklist for DSM-5 and then select which event was the *worst* event that they would report on in the PTSD Checklist for DSM-5 (PCL-5) [82]. The PCL-5 asks patients to report how bothered they have been by 20 symptoms consistent with the *DSM-5* diagnostic criteria for PTSD, with item responses ranging from 0 (not at all) to 4 (extremely). The items are summed so that higher scores indicate greater severity of PTSD symptoms. The PCL-5 has demonstrated good internal consistency, test-retest reliability, and convergent and discriminant validity. A cutoff score between 31 and 33 indicates clinically significant symptoms.

Patients will be asked about alcohol and substance use using the Alcohol, Smoking, and Substance Involvement Screening Test (ASSIST) developed for the World Health Organization. The ASSIST has demonstrated good concurrent, construct, and discriminative validity, as well as test-retest reliability [83,84]. Patients will report on alcohol and nonprescription drug use at each time point. The ASSIST includes 8 questions to assess use frequency, severity, and related problems. Items are summed so that higher scores indicate a greater risk of problems associated with use and cutoff scores that indicate low-, moderate-, and high-risk stratification.

### Plan of Analysis

Regarding the longitudinal, observational study of patients, quantitative data will be viewed graphically and analyzed descriptively. Qualitative open-ended responses to questions will be summarized into common themes or content analyzed, as appropriate. The rates of recruitment of at-risk patients will inform the sample size needed and length of data collection periods for a full-scale trial. Feasibility of retention will be indicated by 80% follow-up rates with participants. We will examine the feasibility of collecting self-report and EHR data linked to patients and the feasibility of locating and pulling EHR data related to suicide-prevention services and outcomes in a data warehouse. Problems with feasibility will be addressed and incorporated into the full-scale trial planning.

## Results

Project WISE was funded in July 2019. As of September 2021, we have completed data collection and preliminary analyses for the focus groups (N=27) and usability testing (N=3). We anticipate that the publication of these findings will be in spring 2022. Workflow observation of nurses on the unit will occur during aims 3 and 4. We will begin enrolling nurses and patients for aims 3 and 4 in November 2021, and we anticipate the completion of data collection by November 2022.

## Discussion

### Principal Findings

Project WISE is a response to the increasing rates of suicide in the United States and calls for health care settings and providers to play a greater role in suicide prevention [5,85]. The findings will inform the development of technology-enhanced training in suicide safety planning and the design and procedures for a fully powered cluster randomized trial to evaluate the impact of this training for nurses on patients’ suicide-related outcomes. In all phases of the research, Project WISE takes a pragmatic approach, both in terms of what is developed and deployed as well as how the impact of the training on patients will be evaluated. There are several pragmatic considerations for a future trial design. This includes planning for a design that randomizes medical, surgical, and trauma units by cluster and the collection of data at the unit level. Although EHR data are highly pragmatic, procedures for collecting these data can vary greatly by health care system and setting [86]. This pilot research will provide critical insight into how best to capture data from the local hospital data warehouse for population-level assessment of service delivery targets and patient outcomes. Patient-reported outcomes are frequently collected alongside EHR or administrative data in pragmatic trials. We will collect patient-reported outcomes in a future trial and will pilot pragmatic web-based longitudinal data collection procedures. This includes using a self-report measure of suicidal ideation and behavior previously used in web-based research and piloting both independent and interview-based completion of surveys. Finally, recruiting patients using the C-SSRS triage version screening data has pragmatic appeal in the study site as nurses will have this information available in routine care with a usual care workflow to address patient safety concerns. We recognize that enhancing usual care by having nurses engage in suicide safety planning may not be readily generalized to other hospitals without the existing infrastructure for suicide risk screening.

Project WISE is situated within a larger movement to improve the population impact of behavioral health care through alternative models of service delivery. The challenges of reaching the full population of people in need and the disparities in received care among racial or ethnic minority populations are well-documented [87,88]. This movement has gained even greater momentum in the recent context of the COVID-19 pandemic, which has led to societal- and individual-level changes such as increased social isolation, unemployment, and fear of contracting the virus, which are expected to result in a greater need at a population level to help with depression, anxiety, substance use problems, and suicidality [89,90]. A service delivery model that Project WISE aligns with is integrated care. Integrated care refers to patients receiving behavioral health and medical care within the same service setting and is known to reduce access and stigma barriers by serving patients where they are already being seen [91]. Consistent with the integrated care model, Project WISE encourages providing some suicide-prevention services in the hospital setting, thereby not requiring patients to make it to outpatient referrals before getting help with suicidality. As many suicidal patients will not make it to outpatient referrals [92], safety planning with their nurse may be the only immediate practical help with suicidality these patients receive.

Project WISE also promotes a task-shifting approach, which moves the intervention delivery to health care workers with fewer mental health care qualifications but who can be trained in a short period to effectively deliver these interventions [93,94]. Task-shifting increases the availability of resources by reducing reliance on specialty providers who are generally costlier and less available. Inpatient medical, surgical, and trauma care nurses are already on the frontlines working closely with patients with behavioral health needs, and there is increasing recognition that nurses want and need additional training to effectively support these patients [12,95]. Engaging patients in suicide safety planning does not require having advanced mental health training, and as a brief intervention, it is particularly well-suited for a task-shifting approach in a hospital setting with nurses. The premise of Project WISE is that nurses who are already screening for suicidality, working closely with patients, and helping patients prepare for discharge from the hospital are ideally positioned—with some additional support and education—to engage patients in safety planning.

Project WISE was born out of research on how to effectively and pragmatically train frontline trauma center providers in brief evidence-based counseling interventions [52,93,96]. From these experiences, it was clear that the gold standard training models developed through implementation science [32] could be challenging to implement in routine care environments and that accessible, flexible, and efficient skill-building approaches are needed. This need led us to harness novel artificial intelligence–based technologies for the skill-building components of the Project WISE training. Advances in computing power have allowed the field of artificial intelligence to flourish in the past 20 years, and the use of artificial intelligence is quickly becoming normative across medicine for a variety of purposes [97,98]. Although these technologies can be costly to develop initially, they can be highly pragmatic and cost-effective for subsequent deployment [99]. The Lyssn Advisor technology is past the initial development phase and is being deployed in outpatient behavioral health settings, with ongoing research to evaluate its impact [39,41]. Advances in study technologies are ongoing. Project WISE technologies currently focus on training in general counseling skills, also known as *common factors*, that are important across counseling or psychological interventions [44]. However, applications of the automated coding technology are now being developed to assess the quality of the unique aspects of interventions, such as cognitive-behavioral techniques, that are believed to also cause change in patients and improvement in patient outcomes [100]. Future development on the quality of suicide-prevention interventions such as safety planning may become available and incorporated into future research.

To ensure that nurses will engage with the Client Bot and Lyssn Advisor technologies, Project WISE incorporates user-centered design principles and methods, such as usability testing. However, the role of user-centered design is integral to the overall approach of the research. In particular, Project WISE aims to integrate the methods, models, and principles from user-centered design and implementation science. Implementation science is the study of processes and strategies that integrate evidence-based interventions into usual care settings, and provider training is one of the common and well-studied implementation strategies [101]. User-centered design is the process of designing products with the involvement of those who will use the product and incorporate their needs and preferences into the design [102]. Both fields are concerned with getting innovations into routine practice and appreciate the importance of the intervention delivery context. However, an important difference is that implementation science has traditionally focused on how to move innovations into routine practice after the innovation has been developed, whereas user-centered design does so during the development process. Project WISE takes the stance that the implementation strategy of training must itself be designed with input from the end user while taking context into consideration. The planned research ensures that nurses’ perspectives and hospital setting constraints are integral to developing and deploying the training.

Although not an explicit focus of Project WISE, findings from implementation science also underscore that training is necessary but insufficient to ensure that providers are able to deliver a high-quality, evidence-based intervention over time, a problem that can be assuaged with occasional feedback on performance or refresher training [32]. The automated coding technology used in Project WISE can be harnessed for this purpose, and future research could examine how to most effectively use the technology specifically for the purpose of managing drift. The problem of drift speaks to the potential need for booster training, and the intention behind the workplace integrated training model of Project WISE is to make such booster sessions readily accessible.

### Conclusions

Project WISE includes developmental and pilot research for a technology-enhanced skills-based training that can be integrated into the routine workflow of nurses to support them in engaging medical, surgical, or traumatically injured inpatients in a brief suicide-prevention intervention. Project WISE is designed to uncover the challenges and opportunities in engaging nurses in e-learning training, as well as the feasibility of a future pragmatic trial. Therefore, the procedures and components of the resulting full-scale trial may look different from those proposed in this pilot research. Regardless, much will be learned about suicide prevention with hospitalized patients, the role of nurses in this work, and how nurses engage with novel training technologies. As such, Project WISE is expected to help further the national agenda of implementing suicide prevention in health care settings and inform best practices for meeting the needs for provider training that will be required to reach this goal.

## Appendix

Multimedia Appendix 1 NIMH Peer-Review Summary Statement.

## Abbreviations

ASSIST: Alcohol, Smoking, and Substance Involvement Screening Test
C-SSRS: Columbia Suicide Severity Rating Scale
DSM-5: Diagnostic and Statistical Manual of Mental Disorders, Fifth Edition
ED: emergency department
EHR: electronic health record
PCL-5: Posttraumatic Stress Disorder Checklist–5
PTSD: posttraumatic stress disorder
SITBI-R: Self-Injurious Thoughts and Behaviors Interview–revised
SUS: System Usability Scale
TDF: Theoretical Domains Framework
WISE: Workplace Integrated Support and Education
