# Supplementary material for: Harnessing Innovative Technologies to Train Nurses in Suicide Safety Planning With Hospitalized Patients: Protocol for Formative and Pilot Feasibility Research
Source: JMIR Res Protoc. 2021 Dec 15;10(12):e33695. doi: 10.2196/33695 (PMC8717131; doi:10.2196/33695)
Supplement: Multimedia Appendix 1 [file resprot_v10i12e33695_app1.pdf]

SUMMARY STATEMENT
(Privileged Communication)

PROGRAM CONTACT:
Lauren Hill
301-443-2638
hillla@mail.nih.gov

Release Date: 03/21/2019
Revised Date:

Application Number: 1 K23 MH118361-01A1

Principal Investigator

DARNELL, DOYANNE ASPEN

Applicant Organization: UNIVERSITY OF WASHINGTON

Review Group: SERV
Mental Health Services Research Committee

Meeting Date: 02/27/2019
Council: MAY 2019
Requested Start: 07/01/2019

RFA/PA: PA18-374
PCC: 8K-RT

Project Title: Technologic Innovation to Enhance the Scalability and Sustainability of Trauma Center Provider Training in Suicide Safety Planning
SRG Action: Impact Score:26
Next Steps: Visit https://grants.nih.gov/grants/next_steps.htm
Human Subjects: 48-At time of award, restrictions will apply
Animal Subjects: 10-No live vertebrate animals involved for competing appl.
Gender: 1A-Both genders, scientifically acceptable
Minority: 1A-Minorities and non-minorities, scientifically acceptable
Children: 3A-No children included, scientifically acceptable

Project Year	Direct Costs Requested	Estimated Total Cost
1	143,236	154,695
2	146,624	158,354
3	146,883	158,634
4	139,923	151,117
TOTAL	576,666	622,799

ADMINISTRATIVE BUDGET NOTE: The budget shown is the requested budget and has not been adjusted to reflect any recommendations made by reviewers. If an award is planned, the costs will be calculated by Institute grants management staff based on the recommendations outlined below in the COMMITTEE BUDGET RECOMMENDATIONS section.

1K23MH118361-01A1 DARNELL, DOYANNE

RESUME AND SUMMARY OF DISCUSSION: In this resubmission of a K23 application, career development and a research plan are proposed for scalable and sustainable training of healthcare providers to deliver suicide prevention interventions. Areas proposed for the candidate's career development include technology-focused team science, implementation science, user-centered design, and acute care suicide prevention clinical trials research. The sequential research design seeks to identify implementation barriers, followed by a user-centered design process to develop a training program for frontline nurses in trauma settings to deliver Suicide Safety Planning Intervention (SPI). In the final aim of the research plan, a pilot randomized trial (n=20 nurses) will compare a technology-enhanced (i.e., conversational agent or Client-bot, and automated quality assessment) provider training versus web-based didactic only condition. The application comes from an outstanding and productive candidate with a clear commitment to research and the patient population. She is supported by a highly accomplished mentoring team, and each member fills a specific niche in support of the candidate's career development. In general, the career development plan is comprehensive and is well linked to the components for the proposed research. Overall, the resubmission is generally responsive to the previous review, in which the feasibility of the research and training plans were questioned. However, both the research and career development plans remain ambitious, and some specific details underspecified (e.g., recruitment of nurses, time needed for intervention delivery in a busy trauma setting). In particular, the analytic plan is insufficiently developed. Despite these minor weaknesses, there is a great deal of enthusiasm for this candidate's career development with this mentoring team and institutional environment, and there is a high likelihood of her launching an independent career based on these proposed activities.

DESCRIPTION (provided by applicant): Over 44,000 people died by suicide in the U.S. in 2016 and national rates continue to increase. The majority of people who died by suicide had contact with the health care system in the year prior to their death. Major hurdles to implementing suicide prevention in healthcare settings include the lack of scalable and sustainable methods for training routine healthcare providers in suicide prevention. Innovations in machine learning and artificial intelligence may overcome these hurdles as it is now possible for technology to assess the quality of provider skill in intervention delivery and provide opportunities for skill acquisition and practice. The candidate's long-term goal is to harness technological advances in artificial intelligence, natural language processing, and machine learning to improve the scalability and sustainability of training among general medical providers in suicide prevention. The proposed research and training activities will take place at the University of Washington at Harborview Medical Center in Seattle, WA, a county safety-net hospital and level I trauma center serving patients across Washington, Wyoming, Alaska, Montana and Idaho. The research aims to adapt and deploy existing scalable technology to train frontline trauma center providers (e.g., nurses) to collaboratively engage patients in a suicide safety planning intervention (SPI) and conduct a pilot feasibility trial of the resultant training. Aim 1 includes focus groups with trauma nurses to identify individual, setting, and organizational-level implementation barriers and facilitators based on the Theoretical Domains Framework and inform strategies for engaging nurses in training and delivery of the SPI with patients. Aim 1 also includes the user-centered design method of contextual inquiry, including task analysis, with nurses to inform workflow- integration. Aim 2 includes user-centered design methods to identify technology refinements and adaptations based on nurse preferences to increase usability. The technologies are a 1) conversational agent, with simulated patient role-play and real-time feedback, and 2) AI-based feedback of counseling performance from SPI audio recordings. Aim 3 is to conduct a pilot randomized trial of a technology-enhanced provider training as compared to a web-based didactic only condition. The longitudinal trial will include 20 nurses (10 per condition), each with 3 patients, and support submission of an NIMH R01 full-scale trial. The K23 training goals include building knowledge and skills in 1) technology-focused team science, 2) the application and integration of implementation science, user-centered design, and adult learning theory for technology adaptation and integration for nurse training, 3) acute care suicide prevention clinical

trials research, including the responsible conduct of research with patients at-risk for suicide, and statistical methods for low base-rate outcomes and nested longitudinal clinical trials data. This K23 application addresses the NIMH Strategic Plan by developing strategies incorporating information technology and pragmatic feedback systems for suicide prevention efforts in real-world practice, reaching the full breadth of patients presenting to the health care system after injury.

PUBLIC HEALTH RELEVANCE

Each year in the United States approximately 30 million individuals require emergency department visits; 1.5- 2.5 million are so severely injured that they require inpatient hospitalization and over 650,000 patients present for self-inflicted injury. Acute care medical settings, such as emergency departments and inpatient trauma units, present critical opportunities for suicide prevention, which is stymied by a lack of scalable and sustainable methods for training providers in high quality suicide prevention interventions. The proposed K23 harnesses advances in artificial intelligence, natural language processing, and machine learning to improve the scalability and sustainability of suicide prevention training among acute medical care providers to better reach the population of patients at-risk for suicide with potentially life-saving interventions.

CRITIQUE 1

Candidate: 2
Career Development Plan/Career Goals /Plan to Provide Mentoring: 3
Research Plan: 3
Mentor(s), Co-Mentor(s), Consultant(s), Collaborator(s): 1
Environment Commitment to the Candidate: 1

Overall Impact:

This second submission of a K23 application that seeks to launch a research career aimed at developing scalable and sustainable training methods for healthcare providers delivering suicide prevention interventions. The three-phase proposed research plan leads to a pilot feasibility trial of a collaboratively developed training program for front-line nurses to deliver suicide safety planning intervention (SPI). The career development plan includes technology-focused team science, SPI and SPI fidelity assessment, and acute care suicide prevention and intervention clinical trials research. The application comes from an outstanding and productive candidate with a clear commitment to research and the patient population. She is supported by a highly accomplished mentoring team – each of whom fulfill a specific niche in support of the candidate's career development. The career development plan addresses the relevant components for the proposed research, and overall, the application seems likely to launch an independent career, with outcomes from the third phase of the research plan intended to inform a full-scale RCT. While the initial review was overall quite enthusiastic of the candidate, career development and plan research aim, the major weakness was the concern that of the research and career development plans were quite ambitious, with many "cutting-edge" elements potentially not feasible in the proposed timeframe. In addition, aspects of the training program and career development plan were not presented with enough detail. The resubmission clarifies that proposed conversational agent ("Client Bot") and automated quality assessment system ("CORE-MI") are both well-validated and ready to use in the clinical setting. Further, the complexity and practical burden of Aim 1 is reduced to focus on the local implementation context as opposed to surveying 25 sites participating in an ongoing multisite clinical trial. There is additional justification for the training plan and add training in implementation science models and methods, as well as provider education and behavior change with an in-depth application of an implementation science framework through self-report nursing surveys during the trial and end-of-trial nurse focus groups. Finally, the resubmission has a reorganization of the training goals around three core areas to better articulate the justification for training activities. The application discusses that this K23 award would not be focused on a new

Suicide Prevention training program, but rather the key innovation is for the candidate to learn how to harness innovative technologies to facilitate skills practice and provide feedback and coaching in an applied context. Overall, the resubmission responds to the major concerns although the career development and research plan is still ambitious.

1. Candidate:
Strengths
- The candidate is a clinical psychologist with a strong prior research training and commitment focused on patient-focused research to improve outcomes in substance abuse and victims of trauma.
- Highly productive with more than 22 publications, has received Diversity Supplement and T-32 award.
- Candidate has strong potential to develop as an independent and productive researcher, as indicated by candidate's prior training and research experiences (including academic productivity to date), letters of reference, and endorsement and support from mentoring/advisor/consultant team.

Weaknesses
- None.

2. Career Development Plan/Career Goals & Objectives:
Strengths
- Career Development Plan and Goals are appropriate for the scientific development of the candidate in the proposed content areas of interest.
- Responded well to previous concerns and didactic plan maps nicely with career aims.

Weaknesses
- Still fairly ambitious goals but somewhat simplified; an implementation goal was added.

3. Research Plan:
Strengths
- Research aims are simplified, with a focus on local implementation instead of surveying 25 sites.
- Better evidence to support the effectiveness of SBI is presented.
- Nursing leader will also be a champion for the project.

Weaknesses
- This is a minor weakness, but it might have strengthened the application to discuss the time needed for each nursing intervention since trauma nurses are very busy at a Level One Trauma center. Exactly when in the visit (inpatient vs ED) the intervention occurs is not clearly specified. The feasibility question will still need to be proven.

4. Mentor(s), Co-Mentor(s), Consultant(s), Collaborator(s):
Strengths
- The mentor/consultant/advisor team appears well positioned to support Dr. Darnell in both her training and research plans, with each team member committing to contribute to a specific aspect or niche content area outlined within her plans.
- Many members of the mentoring team have histories of collaborating with one another, and some have previously worked with Dr. Darnell directly.

Weaknesses
- The number of mentors and advisors will require significant program coordination and time. The mentoring plan does not appear to be significantly simplified in the resubmission.

5. Environment and Institutional Commitment to the Candidate:

Strengths

- Strong environment (institutional and departmental support were clear) with resources necessary to support Dr. Darnell's training and research plan. Training and research plan aligns with current departmental initiatives and, more broadly, the university focus on population health.
- Harborview Medical Center, the host site for this project, is home to a Level 1 Trauma Center managed by the University of Washington School of Medicine and sees 5,500-6,500 trauma admissions per year.

Weaknesses

- None.

Study Timeline:
Strengths

- Reasonable timeline.

Weaknesses

- None.

Protections for Human Subjects:
Acceptable Risks and Adequate Protections
Data and Safety Monitoring Plan (Applicable for Clinical Trials Only):
Acceptable.

Inclusion of Women, Minorities and Children:

- Sex/Gender: Distribution justified scientifically
- Race/Ethnicity: Distribution justified scientifically
- For NIH-Defined Phase III trials, Plans for valid design and analysis: Not applicable
- Inclusion/Exclusion of Children under 18: Excluding ages <18; justified scientifically

Training in the Responsible Conduct of Research:
Acceptable
Comments on Format (Required):

- Acceptable.

Comments on Subject Matter (Required):

- Acceptable.

Comments on Faculty Participation (Required; not applicable for mid- and senior-career awards):

- Acceptable.

Comments on Duration (Required):

- Acceptable.

Comments on Frequency (Required):

- Acceptable.

Authentication of Key Biological and/or Chemical Resources:
Not Applicable (No Relevant Resources)

CRITIQUE 2

Candidate: 1
Career Development Plan/Career Goals /Plan to Provide Mentoring: 2
Research Plan: 4
Mentor(s), Co-Mentor(s), Consultant(s), Collaborator(s): 1

Environment Commitment to the Candidate: 1

Overall Impact:
This resubmission of a K23 application is from a qualified candidate who is surrounded by a qualified team of mentors and consultants. The application is somewhat responsive to the prior review. Some of the proposed training is online – in particular, the natural language processing and machine learning. The machine learning will be complex, and it is not fully clear if this is necessary for the proposed training, since the CORE-MI and Client-Bot already are developed and only the output will be used in this research. The study procedures for Aims 1-3 lack adequate details about the timing of assessments. The analytic plan seems to be one small paragraph, which also lacks adequate details and rigor. Similar concerns were raised in the prior submission but did not seem to be adequately addressed in the current submission.

1. Candidate:
Strengths
- The candidate has been very productive as noted by her publications.
- The K23 application builds upon a solid foundation in psychology and mental health services research.

Weaknesses
- [None noted]

2. Career Development Plan/Career Goals & Objectives:
Strengths
- The training goals of technology-focused training, implementation science, and suicide prevention clinical trials are appropriate.
- The training is tied to the research.

Weaknesses
- It is not fully clear that auditing the biostatistics course would be sufficient, and it is not clearly described who the mentor would be to advise the candidate when implementing these analytic methods.
- The machine learning online course is probably not the right course for the proposed research. Machine learning is a very technical application, and it does not seem that this will be used in the proposed research.

3. Research Plan:
Strengths
- The first two aims are well positioned to understand how to best integrate the technology in practice.
- The use of existing technologies – CORE-MI and Client Bot – is a strength. These have been used and the candidate has experienced mentors to assist her with implementation of this technology.

Weaknesses
- The research plan lacks detail of how and where nurses will be recruited or any anticipated response rate. Much of the details appear to be embedded in the Human Subjects Section. At least 30 nurses will participate in Aim 1, 3 in Aim 2, and 20 in Aim 3, with those participating in Aim 3 naïve to the study. It might have strengthened the application to discuss what is the base pool from which the trauma nurses are going to be recruited and how likely it will be to recruit the desired sample.
- The feasibility of organizing 3 focus groups of 10 nurses each is not adequately discussed. The challenge is getting a time and location where all can attend.
- The setting from which the nurses and patients will be recruited is not fully described, and there is insufficient discussion about the study setting and where study procedures will take place.

- The timing of the assessments for Aim 3 appears to be baseline, 1 month and 6 months. Patients' complete assessments at 1 and 3 months, and there are patient encounter recordings that will happen soon after the standardized patient assessment. There are a lot of moving parts of this trial, and it is not fully clear how data collection is organized and mapped out.
- It appears that patients are recruited during the inpatient stay, and all study procedures are completed before discharge (these individuals are in the facility for 3 months). The Human Subjects form has more detail and states that patients complete all study procedures prior to discharge. Without information about this particular setting, it is difficult to place this in context with respect to a patient's length of stay, etc. On page 174, there is mention of contact numbers for follow up, so some individuals presumably will leave the facility.
- There seems to be nothing mentioned about any incentives for participants. The same can be said for the nurses.
- The research assistant will require substantial training, but it is not fully clear when and who will do this.
- There is limited discussion about the possibility for missing data and how the candidate will handle this.
- The data analysis plan seems particularly sparse. It is not clearly described how all of the assessments collected during the randomized trial will be operationalized and entered into the analytic models and whether the main outcome is baseline to 6 months for the nurses and baseline to 3 months for the patients.
- The end of study focus group is mentioned, but there is insufficient detail about this component of the study, who will participate, and how it will be structured and analyzed.

4. Mentor(s), Co-Mentor(s), Consultant(s), Collaborator(s):
Strengths
- The mentors and consultants are accomplished researchers with expertise in the key areas of the training and research plan.

Weaknesses
- [None noted]

5. Environment and Institutional Commitment to the Candidate:
Strengths
- The institutional environment is strong.

Weaknesses
- [None noted]

Study Timeline:
Strengths
- The clinical trial is designed as a feasibility and acceptability pilot, and it is adequately over a year and one-half to recruit 20 nurses and 3 of their patients.

Weaknesses
- [None noted]

Protections for Human Subjects:
Unacceptable Risks and/or Inadequate Protections
- Recruitment of patients at the bedside may breach confidentiality, since these are not private rooms. The application does not adequately discuss what would be done to minimize this.

Data and Safety Monitoring Plan (Applicable for Clinical Trials Only):
Unacceptable.
  - A DSMP does not seem to be included in the application.

Inclusion of Women, Minorities and Children:

- Sex/Gender: Distribution justified scientifically
- Race/Ethnicity: Distribution justified scientifically
- For NIH-Defined Phase III trials, Plans for valid design and analysis: Not applicable
- Inclusion/Exclusion of Children under 18: Excluding ages <18; justified scientifically
- Inclusion and exclusions justified.

Vertebrate Animals:
Not Applicable (No Vertebrate Animals).

Biohazards:
Not Applicable (No Biohazards).

Training in the Responsible Conduct of Research:
Acceptable.
Comments on Format (Required):
- Format is varied and adequate.

Comments on Subject Matter (Required):
- Subject matter is comprehensive

Comments on Faculty Participation (Required; not applicable for mid- and senior-career awards):
- Preceptors are involved in the training.

Comments on Duration (Required):
- The duration is adequate

Comments on Frequency (Required):
- Frequency is also adequate and meets standards.

Select Agents:
Not Applicable (No Select Agents).

Resource Sharing Plans:
Not Applicable (No Relevant Resources).

Authentication of Key Biological and/or Chemical Resources:
Not Applicable (No Relevant Resources).

CRITIQUE 3

Candidate: 2
Career Development Plan/Career Goals /Plan to Provide Mentoring: 2
Research Plan: 3
Mentor(s), Co-Mentor(s), Consultant(s), Collaborator(s): 2
Environment Commitment to the Candidate: 1

Overall Impact:
The application outlines major hurdles to scalable and sustainable methods for training routine healthcare providers in suicide prevention. Innovative technologies include conversational agent and AI-based feedback of counseling performance. This K23 application will utilize computer technology for the purposes of training general medical providers in suicide prevention and monitoring the quality of skills ongoing. The candidate is a clinical psychologist that has focused on patient-centered research to improve outcomes in substance abuse and victims of trauma. She has been very productive and is well situated for a K23 patient-oriented career award. The application documents the need for training

for medical personnel in suicide prevention. The training plan includes certification in User-Centered Design, two online courses to gain basic knowledge of the computer technology underlying the Client Bot and CORE-MI technologies, and two courses at the University of Washington on Dissemination and Implementation Science Clinical Trials Biostatistics. The mentorship team provides a good supportive structure and expertise to meet the proposed training and research goals. The research aims include the development of a technology-based training for a Suicide Safety Planning Intervention (SPI) using a conversational agent and compare it to a web-based didactic only. This resubmission is responsive to the previous review regarding feasibility of the research and training plans.

1. Candidate:
Strengths
- The candidate is a clinical psychologist that has focused on patient-centered research to improve outcomes in substance abuse and victims of trauma. She has been very productive and is well situated for a K23 patient-oriented career award.

Weaknesses
- None.

2. Career Development Plan/Career Goals & Objectives:
Strengths
- Includes certification in User-Centered Design, two online courses to gain basic knowledge of the computer technology underlying the Client Bot and CORE-MI technologies, and two courses at the University of Washington on Dissemination and Implementation Science Clinical Trials Biostatistics.

Weaknesses
- None.

3. Research Plan:
Strengths
- Innovative technologies include conversational agent and AI-based feedback of counseling performance.
- Responsive to previous review in regard to feasibility.

Weaknesses
- Limited analytic plan.

4. Mentor(s), Co-Mentor(s), Consultant(s), Collaborator(s):
Strengths
- The mentorship team provides a good supportive structure and expertise to meet the proposed training and research goals.

Weaknesses
- None.

5. Environment and Institutional Commitment to the Candidate:
Strengths
- Outstanding.

Weaknesses
- None.

Study Timeline:
Strengths
- [None noted.]

Weaknesses
- [None noted.]

Protections for Human Subjects:
Acceptable Risks and Adequate Protections
Data and Safety Monitoring Plan (Applicable for Clinical Trials Only):
Acceptable

Inclusion of Women, Minorities and Children:
- Sex/Gender: Distribution justified scientifically
- Race/Ethnicity: Distribution justified scientifically
- For NIH-Defined Phase III trials, Plans for valid design and analysis:
- Inclusion/Exclusion of Children under 18: Excluding ages <18; justified scientifically

Training in the Responsible Conduct of Research:
Acceptable
Comments on Format (Required):
Comments on Subject Matter (Required):
Comments on Faculty Participation (Required; not applicable for mid- and senior-career awards):
Comments on Duration (Required):
Comments on Frequency (Required):

THE FOLLOWING SECTIONS WERE PREPARED BY THE SCIENTIFIC REVIEW OFFICER TO SUMMARIZE THE OUTCOME OF DISCUSSIONS OF THE REVIEW COMMITTEE, OR REVIEWERS' WRITTEN CRITIQUES, ON THE FOLLOWING ISSUES:

PROTECTION OF HUMAN SUBJECTS: UNACCEPTABLE. In Study 4, recruitment of patients at the bedside may breach confidentiality, since these are not private rooms. The application does not adequately discuss what would be done to minimize this.

INCLUSION OF WOMEN PLAN: ACCEPTABLE

INCLUSION OF MINORITIES PLAN: ACCEPTABLE

INCLUSION OF CHILDREN PLAN: ACCEPTABLE. Children are appropriately excluded.

COMMITTEE BUDGET RECOMMENDATIONS: The budget was recommended as requested.

Footnotes for 1 K23 MH118361-01A1; PI Name: Darnell, Doyanne Aspen

NIH has modified its policy regarding the receipt of resubmissions (amended applications). See Guide Notice NOT-OD-14-074 at http://grants.nih.gov/grants/guide/notice-files/NOT-OD-14-074.html. The impact/priority score is calculated after discussion of an application by averaging the overall scores (1-9) given by all voting reviewers on the committee and multiplying by 10. The criterion scores are submitted prior to the meeting by the individual reviewers assigned to an application, and are not discussed specifically at the review meeting or calculated into the overall impact score. Some applications also receive a percentile ranking. For details on the review process, see http://grants.nih.gov/grants/peer_review_process.htm#scoring.

MEETING ROSTER

Mental Health Services Research Committee
National Institute of Mental Health Initial Review Group
NATIONAL INSTITUTE OF MENTAL HEALTH
SERV
02/27/2019

Notice of NIH Policy to All Applicants: Meeting rosters are provided for information purposes only. Applicant investigators and institutional officials must not communicate directly with study section members about an application before or after the review. Failure to observe this policy will create a serious breach of integrity in the peer review process, and may lead to actions outlined in NOT-OD-14-073 at https://grants.nih.gov/grants/guide/notice-files/NOT-OD-14-073.html and NOT-OD-15-106 at https://grants.nih.gov/grants/guide/notice-files/NOT-OD-15-106.html, including removal of the application from immediate review.

CHAIRPERSON(S)

STUART, ELIZABETH A., PHD
PROFESSOR
DEPARTMENT OF MENTAL HEALTH
DEPARTMENT OF BIOSTATISTICS
JOHNS HOPKINS BLOOMBERG SCHOOL OF PUBLIC HEALTH
BALTIMORE, MD 21205

MEMBERS

AHMEDANI, BRIAN K., PHD
ASSOCIATE SCIENTIST
CENTER FOR HEALTH POLICY & HEALTH SERVICES
RESEARCH, BEHAVIORAL HEALTH SERVICES
HENRY FORD HEALTH SYSTEM
DETROIT, MI 48202

BAKER-ERICZEN, MARY J, PHD *
RESEARCH SCIENTIST
CHILD AND ADOLESCENT SERVICES RESEARCH CENTER
OPERATIONS DIRECTOR
RADY CHILDREN'S HOSPITAL, SAN DIEGO
SAN DIEGO, CA 92123

BELLAMY, CHYRELL DENISE, PHD *
ASSOCIATE PROFESSOR
DIRECTOR OF PEER SERVICES AND RESEARCH
PROGRAM FOR RECOVERY AND COMMUNITY HEALTH
DEPARTMENT OF PSYCHIATRY
YALE SCHOOL OF MEDICINE
NEW HAVEN, CT 06513

BENNETT, IAN MOORE, PHD *
PROFESSOR
UNIVERSITY OF WASHINGTON
DEPARTMENT OF GLOBAL HEALTH
SEATTLE, WA 98195

BRESLAU, JOSHUA A, PHD *
SENIOR RESEARCHER
RAND CORPORATION
PITTSBURG, PA 15213

BRUCKNER, TIM ALLEN, PHD *
ASSOCIATE PROFESSOR
DEPARTMENT OF PUBLIC HEALTH
UNIVERSITY OF CALIFORNIA, IRVINE
IRVINE, CA 92697

CHACKO, ANIL, PHD *
ASSOCIATE PROFESSOR
DEPARTMENT OF APPLIED PSYCHOLOGY
NEW YORK UNIVERSITY
NEW YORK, NY 10003

CHILDRESS, DEBRA, PHD *
SENIOR RESEARCH ASSOCIATE
3-C INSTITUTE FOR SOCIAL DEVELOPMENT
DURHAM, NC 27513

COHEN, DEBORAH JILL, PHD
PROFESSOR
SCHOOL OF FAMILY MEDICINE
DEPARTMENT OF FAMILY MEDICINE
OREGON HEALTH AND SCIENCE UNIVERSITY
PORTLAND, OR 97213

COHEN, TREVOR, PHD *
PROFESSOR
SCHOOL OF BIOMEDICAL INFORMATICS
HEALTH SCIENCE CENTER
UNIVERISTY OF WASHINGTON, SEATTLE
SEATTLE, WA 98195

COOK, BENJAMIN LE, PHD
DIRECTOR
CENTER FOR MULTICULTURAL MENTAL HEALTH RESEARCH
DEPARTMENT OF PSYCHIATRY
CAMBRIDGE HEALTH ALLIANCE
CAMBRIDGE, MA 02141

DE CHOUDHURY, MUNMUN, PHD *
ASSISTANT PROFESSOR
SCHOOL OF INTERACTIVE COMPUTING
GEORGIA INSTITUTE OF TECHNOLOGY
ATLANTA, GA 30332

DOSREIS, SUSAN C, PHD
PROFESSOR
DEPARTMENT OF PHARMACEUTICAL HEALTH SERVICES
UNIVERSITY OF MARYLAND SCHOOL OF PHARMACY
BALTIMORE, MD 21201

DRAHOTA, AMY, PHD *
ASSISTANT PROFESSOR
MICHIGAN STATE UNIVERSITY
CLINICAL PSYCHOLOGY AND ECOLOGICAL
COMMUNITY PSYCHOLOGY PROGRAM
DEPARTMENT OF PSYCHOLOGY
EAST LANSING, MI 48824

EISENBERG, DANIEL, PHD
S.J. AXELROD COLLEGIATE PROFESSOR
DEPARTMENT OF HEALTH MANAGEMENT AND POLICY
SCHOOL OF PUBLIC HEALTH
UNIVERSITY OF MICHIGAN
ANN ARBOR, MI 48109

FEIL, EDWARD GUSTAV, PHD *
RESEARCH SCIENTIST
OREGON RESEARCH INSTITUTE
EUGENE, OR 97403

FELTON, MICHELE
PROGRAM COODINATOR
WASHINGTON UNIVERSITY IN ST. LOUIS
SITEMAN CANCER CENTER
WASHINGTON UINVERSITY
ST. LOUIS, MO 63110

FIELD, ALISON E *
PROFESSOR AND CHAIR
DEPARTMENT OF EPIDEMIOLOGY
BROWN UNIVERSITY SCHOOL OF PUBLIC HEALTH
PROVIDENCE, RI 02912

GLADSTONE, TRACY G, PHD *
SENIOR RESEARCH SCIENTIST
WELLESLEY COLLEGE
WELLESLEY CENTERS FOR WOMEN
CHESTNUT HILL, MA 02481

GOODKIND, JESSICA R, PHD *
ASSOCIATE PROFESSOR
DEPARTMENTS OF SOCIOLOGY
UNIVERSITY OF NEW MEXICO
ALBUQUERQUE, NM 87131

GRUPP-PHELAN, JACQUELINE M., MD
PROFESSOR AND CHIEF
PROFESSOR OF CLINICAL EMERGENCY MEDICINE, STEP 4
CHIEF, DIVISION OF PEDIATRIC EMERGENCY MEDICINE
DEPARTMENT OF EMERGENCY MEDICINE
UNIVERSITY OF CALIFORNIA, SAN FRANCISCO
SAN FRANCISCO, CA 94143

HARTWELL, STEPHANIE W, PHD *
PROFESSOR OF SOCIOLOGY
COLLEGE OF LIBERAL ARTS & SCIENCE
CLAS DEAN'S OFFICE
WAYNE STATE UNIVERSITY
DETROIT, MI 48201

HERSCHELL, AMY D, PHD
SENIOR DIRECTOR
RESEARCH STRATEGY AND OUTCOMES MANAGEMENT
COMMUNITY CARE BEHAVIORAL HEALTH ORGANIZATION
PITTSBURGH, PA 15222

HOLDEN, RICHARD J, PHD *
ASSOCIATE PROFESSOR OF MEDICINE
INDIANA UNIVERSITY SCHOOL OF MEDICINE
DIVISION OF GENERAL INTERNAL MEDICINE & GERIATRICS
INDIANAPOLIS, IN 46202

JENSEN-DOSS, AMANDA, PHD
ASSOCIATE PROFESSOR
DEPARTMENT OF PSYCHOLOGY
COLLEGE OF ARTS AND SCIENCES
UNIVERSITY OF MIAMI
CORAL GABLES, FL 33124

KARVER, MARC STUART, PHD *
ASSOCIATE PROFESSOR
UNIVERSITY OF SOUTH FLORIDA
PSYCHOLOGY DEPT.
COLLEGE OF ARTS AND SCIENCES
TAMPA, FL 33620

KIENE, SUSAN MARIA, PHD *
PROFESSOR
DIVISION OF EPIDEMIOLOGY AND BIOSTATISTICS
SCHOOL OF PUBLIC HEALTH
SAN DIEGO STATE UNIVERSITY
SAN DIEGO, CA 92182

KLOOS, BRET R, PHD *
ASSOCIATE PROFESSOR
DEPARTMENT OF PSYCHOLOGY
UNIVERSITY OF SOUTH CAROLINA
COLUMBIA, SC 29205

KRATCHMAN, AMY
DIRECTOR OF FAMILY COLLABORATION
FAMILY COLLABORATION
THE CHILDREN'S HOSPITAL OF PHILADELPHIA
PHILADELPHIA, PA 19104

LITZ, BRETT T., PHD *
PROFESSOR
VA BOSTON HEALTHCARE SYSTEM
PROFESSOR
DEPARTMENT OF PSYCHIATRY
BOSTON UNIVERSITY SCHOOL OF MEDICINE
BOSTON, MA 02130

MAZZEO, SUZANNE E, PHD *
PROFESSOR
DEPARTMENT OF PSYCHOLOGY
VIRGINIA COMMONWEALTH UNIVERSITY
RICHMOND, VA 23284

MEEKS, SUZANNE, PHD *
PROFESSOR AND CHAIR
DEPARTMENT OF PSYCHOLOGICAL AND BRAIN SCIENCES
UNIVERSITY OF LOUISVILLE
LOUISVILLE, KY 40292

MEFFERT, SUSAN, MD *
ASSOCIATE PROFESSOR
UNIVERSITY OF CALIFORNIA SAN FRANCISCO
DEPARTMENT OF PSYCHIATRY
UCSF GLOBAL HEALTH SCIENCES FACULTY AFFILIATE
SAN FRANCISCO, CA 941143

MORALES, KNASHAWN HODGE
ASSOCIATE PROFESSOR OF BIOSTATISTICS
DEPARTMENT OF BIOSTATISTICS, EPIDEMIOLOGY, AND INFORMATICS
PERELMAN SCHOOL OF MEDICINE
UNIVERSITY OF PENNSYLVANIA
PHILADELPHIA, PA 19104

NIENDAM, TARA ANN, PHD *
ASSSCIATE PROFESSOR
UCDHS: PSYCHIATRY AND BEHAVIORAL SCIENCES
DEPT. OF IMAGING RESEARCH CENTER/UCDMC
SACRAMENTO, CA 95817

RUSSINOVA, ZLATKA, PHD *
DIRECTOR OF RESEARCH
BOSTON UNIVERSITY
CENTER FOR PSYCHIATRIC REHABILITATION
BOSTON, MA 02215

SALZER, MARK S., PHD
PROFESSOR
DEPARTMENT OF REHABILITATION SCIENCES
COLLEGE OF PUBLIC HEALTH
TEMPLE UNIVERSITY
PHILADELPHIA, PA 19122

SIDDIQUE, JUNED
ASSOCIATE PROFESSOR
PREVENTIVE MEDICINE
FEINBURG SCHOOL OF MEDICINE
NORTHWESTERN UNIVERSITY
CHICAGO, IL 60611

SLEATH, BETSY LYNN, PHD
GEORGE H COCOLAS DISTINGUISHED PROFESSOR AND CHAIR
PHARMACEUTICAL OUTCOMES AND POLICY
SCHOOL OF PHARMACY
UNIVERSITY OF NORTH CAROLINA AT CHAPEL HILL
CHAPEL HILL, NC 27599

SMITH, THOMAS E, PHD *
SPECIAL LECTURER IN PSYCHIATRY
COLUMBIA UNIVERSITY
NEW YORK STATE OFFICE OF MENTAL HEALTH
NEW YORK CITY, NY 10027

WATSON, AMY C, PHD *
PROFESSOR
JANE ADDAMS COLLEGE OF SOCIAL WORK
UNIVERSITY OF ILLINOIS AT CHICAGO
CHICAGO, IL 60607

WILSON, AMY BLANK, PHD *
ASSISTANT PROFESSOR
THE UNIVERSITY OF NORTH CAROLINA AT CHAPEL HILL
SW-SCHOOL OF SOCIAL WORK
CHAPEL HILL, NC 27599

WOLFF, JENNIFER CHRISTINE, PHD *
ASSISTANT PROFESSOR
DEPARTMENT OF PSYCHIATRY AND HUMAN BEHAVIOR
BROWN MEDICAL SCHOOL
PROVIDENCE, RI 02903

YOUNG, ALEXANDER S., MD
PROFESSOR
MIRECC, LOS ANGELES VA MEDICAL CENTER
DEPARTMENT OF PSYCHIATRY
UNIVERSITY OF CALIFORNIA-LOS ANGELES
LOS ANGELES, CA 90073

SCIENTIFIC REVIEW OFFICER

SCHULTE, AILEEN, PHD
SCIENTIFIC REVIEW OFFICER
DIVISION OF EXTRAMURAL ACTIVITIES
NATIONAL INSTITUTE OF MENTAL HEALTH
NATIONAL INSTITUTES OF HEALTH
BETHESDA, MD 20892

EXTRAMURAL SUPPORT ASSISTANT

WORTHAM, LYCURTIS
PROGRAM SPECIALIST
DIVISION OF EXTRAMURAL ACTIVITIES
NATIONAL INSTITUTE OF MENTAL HEALTH
NATIONAL INSTITUTES OF HEALTH
BETHESDA, MD 20892

* Temporary Member. For grant applications, temporary members may participate in the entire meeting or may review only selected applications as needed.

Consultants are required to absent themselves from the room during the review of any application if their presence would constitute or appear to constitute a conflict of interest.
